# When the Cure Becomes the Problem: A Case Report of Immune Reconstitution Inflammatory Syndrome Associated With Varicella Encephalitis

**DOI:** 10.7759/cureus.44223

**Published:** 2023-08-27

**Authors:** Kuan-Yu Lin, Adam Streicher, Joseph Wheeler

**Affiliations:** 1 Medical School, Saint Louis University School of Medicine, St. Louis, USA; 2 Internal Medicine, SSM Health Saint Louis University Hospital, St. Louis, USA

**Keywords:** infectious disease medicine, antiretroviral therapies, immune reconstitution syndrome, varicella encephalitis, hiv aids

## Abstract

Immune reconstitution inflammatory syndrome (IRIS) describes a constellation of inflammatory symptoms that develop following the initiation of antiretroviral therapy (ART) in patients with advanced human immunodeficiency virus (HIV). Here, we present a case of a 39-year-old male-to-female transgender patient with advanced HIV who was started on ART during a hospitalization for acute encephalopathy due to a combination of methicillin-resistant *Staphylococcus aureus* (MRSA) meningitis and varicella encephalitis. After adequate treatment of these infections and five weeks after initiation of ART, she developed inflammatory symptoms of malaise, fever, and tachycardia, as well as laboratory findings of leukocytosis consistent with an inflammatory process. Infectious workup did not reveal any evidence of a new infection, and no other undiagnosed inflammatory processes were discovered to explain these symptoms. A diagnosis of IRIS was suspected, possibly induced by a prior varicella infection. Diagnosis of IRIS can be difficult due to heterogeneous symptoms, differing etiologies, variable patient presentations, and the lack of universal diagnostic criteria. As instances of IRIS are not uncommon in patients with a low CD4 count who start on ART, there should be a high index of suspicion when patients present with inflammatory symptoms after initiation of ART. With increased recognition of the disease and improved standardization of diagnostic criteria, more could be understood about the underlying disease process which may allow for better targeted therapies and individualized treatments for patients who develop the immune reconstitution inflammatory syndrome.

## Introduction

Immune reconstitution inflammatory syndrome (IRIS) typically presents in patients with advanced human immunodeficiency virus (HIV) who develop findings consistent with a hyper-inflammatory process (fevers, chills, leukocytosis, fatigue) within a predictable time period after starting antiretroviral therapy (ART) [1-6]. The prevalence of this syndrome in patients who respond to ART has been noted to be 25%, while the overall prevalence of the syndrome was estimated at 10% in those initiated on ART [2,3]. It is more common in those with CD4 count < 50 prior to starting on ART, and typically occurs within two months of ART therapy initiation and while CD4 cell counts are increasing [2,3]. Although there is no consensus on the diagnostic criteria for IRIS, most suggested diagnostic criteria include the following features: presence of acquired immunodeficiency syndrome (AIDS) with a low initial CD4 count (<100) (except for patients with tuberculosis), a positive virologic and immunologic response to ART (as evidenced by a decrease in viral load after initiation of ART), absence of untreated infection or adverse drug reaction, the presence of inflammatory symptoms, and lastly, a temporal association between ART initiation and the onset of clinical illness [7]. 

In this report, we present the case of a 39-year-old patient with advanced HIV who was started on ART for acute encephalopathy and developed inflammatory symptoms five weeks after the start of ART, and who was suspected with IRIS and managed successfully.

## Case presentation

A 39-year-old male-to-female transgender patient with advanced HIV, who was native to Missouri, was transferred to our hospital for acute encephalopathy with associated seizure. She had not been on ART prior to this admission. She presented with a CD4 count of 8 cells/ul and an HIV viral load of 3,280,000 copies/ml, as well as mild leukopenia and normocytic anemia. A comprehensive infectious workup was pursued upon admission (Table 1). The Infectious Disease and Neurology services were consulted, and she was initiated on bictegravir, emtricitabine, and tenofovir alafenamide.

**Table 1:** Pertinent laboratory test results at admission, at time of suspected IRIS, and after resolution of inflammatory symptoms *Histoplasmosis and Blastomyces were tested due to the patient being native to Missouri MCV: mean corpuscular volume; Na: sodium; Cr: creatinine; Ca: calcium; AST: aspartate aminotransferase; MRSA: methicillin-resistant *Staphylococcus aureus;* AFB: acid-fast bacillus; VZV: varicella-zoster virus; BAL: bronchioalveolar lavage; PJP: *Pneumocystis jirovecii* pneumonia; CMV: cytomegalovirus; PCR: polymerase chain reaction; TSH: thyroid stimulating hormone;  IRIS: immune reconstitution inflammatory syndrome; COVID-19: coronavirus disease 2019

		At the time of admission	At the time of suspected IRIS	After resolution of inflammatory symptoms
Complete blood count	WBC	33,000 (86% neutrophils)	173,000 (88% neutrophils)	80,000
	Hemoglobin	7.3 (MCV 84)	10.4 (MCV 96)	9.6 (MCV 92)
	Platelet	172	454	444
Lactate		-	2.5 (H)	0.9
HIV viral load		3,280,000	-	-
CD4 count		8 (1%)	-	-
Pertinent chemistry data	Na (136-146 mEq/L)	133 (L)	147 (H)	140
	Cr (0.6-1.2 mg/dL)	0.66	0.85	0.58
	Ca (8.4-10.2 mg/dL)	8.9	10.3 (H)	10
	AST (12-38 U/L)	42 (H)	70 (H)	27
Respiratory panel		+adenovirus and COVID-19	+ COVID-19	-
CSF studies	Culture	+MRSA	-	-
	AFB	negative		
	Fungal	negative		
	Meningitis panel	+VZV		
Blood culture data	Culture	x2 negative	x2 negative	-
	AFB	negative	negative	
	Fungal	negative	negative	
	Anaerobic	negative	negative	
BAL results (including PJP, fungus, AFB, bacterial, viral, toxo, CMV, mucorales)		negative	negative	-
Other miscellaneous workup		Legionella urine antigen negative, Strep pneumo urine antigen negative, Blastomyces urine antigen negative, Toxoplasma PCR negative, Quantiferon gold negative x2, +Giardia via stool testing	Beta-glucan negative, Galactomannan negative, Urinalysis - noninfectious, TSH: 1.38 (within normal limits)	-

**Figure 1:**
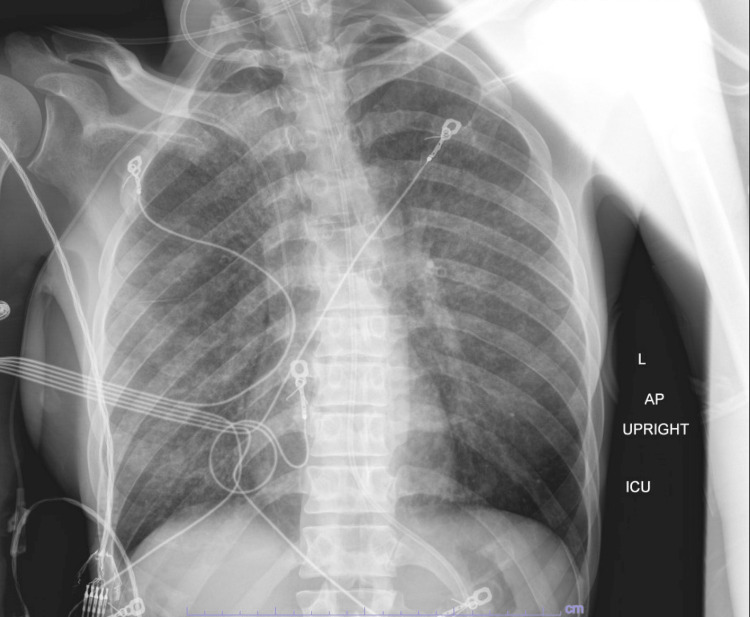
Chest X-ray at admission showing diffuse bilateral pulmonary infiltrate

Initial diagnostic testing was significant for coronavirus disease 2019 (COVID-19) and adenovirus positivity on respiratory viral panel testing. The patient was treated for COVID-19 at an outside hospital. Chest X-ray showed diffuse bilateral pulmonary infiltrates (Figure 1). She underwent lumbar puncture; bacterial cultures from the outside hospital were positive for methicillin-resistant *Staphylococcus aureus* (MRSA) and polymerase chain reaction (PCR) testing was positive for varicella-zoster virus (VZV) in the cerebral spinal fluid (CSF). The patient was initially treated with broad-spectrum antimicrobials, which were narrowed to vancomycin and acyclovir after culture results and susceptibilities were obtained. Testing for a vast array of other opportunistic pathogens in the CSF was negative.

**Figure 2:**
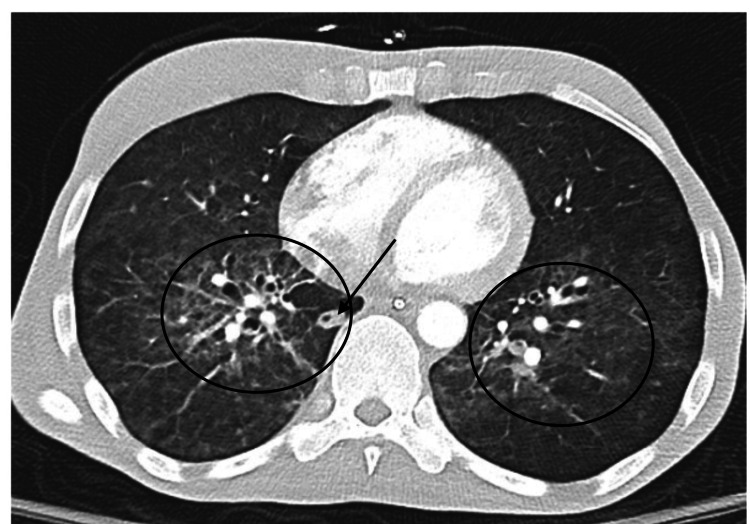
CT of chest at admission showing bilaterally ground-glass opacities and septal thickening (circles) with central cavitation (arrow)

A computerized tomography (CT) scan of her chest was obtained which showed diffuse bilateral ground-glass opacities and septal thickening within the bilateral lungs as well as a likely incidental 3.7 x 9 mm soft tissue nodular opacity with central cavitation within the medial right lower lobe (thought to be bronchial wall thickening versus pulmonary nodule) (Figure 2). Trimethoprim-sulfamethoxazole was started at therapeutic dosing due to concern for *Pneumocystis jirovecii* pneumonia (PJP). A bronchioalveolar lavage (BAL) was obtained and negative for all pathogens including PJP and other opportunistic pathogens; BAL cultures including acid-fast bacilli (AFB) cultures were positive for only *Candida,* which was thought likely to be a contaminant. Given these BAL findings, the findings on the CT chest were thought most likely due to COVID-19 pneumonia, which subsequently improved symptomatically and trimethoprim-sulfamethoxazole was reduced to prophylactic dosing. The patient later developed persistent diarrhea and tested positive for *Giardia* for which she was treated with 10 days of metronidazole. The patient’s mental status did improve; however, it did not return to her previous baseline. This new chronic encephalopathy was thought likely secondary to irreversible effects of the aforementioned central nervous system infections with possible contributions from her end-stage HIV, and a joint decision was made for placement at a skilled nursing facility. 

**Figure 3:**
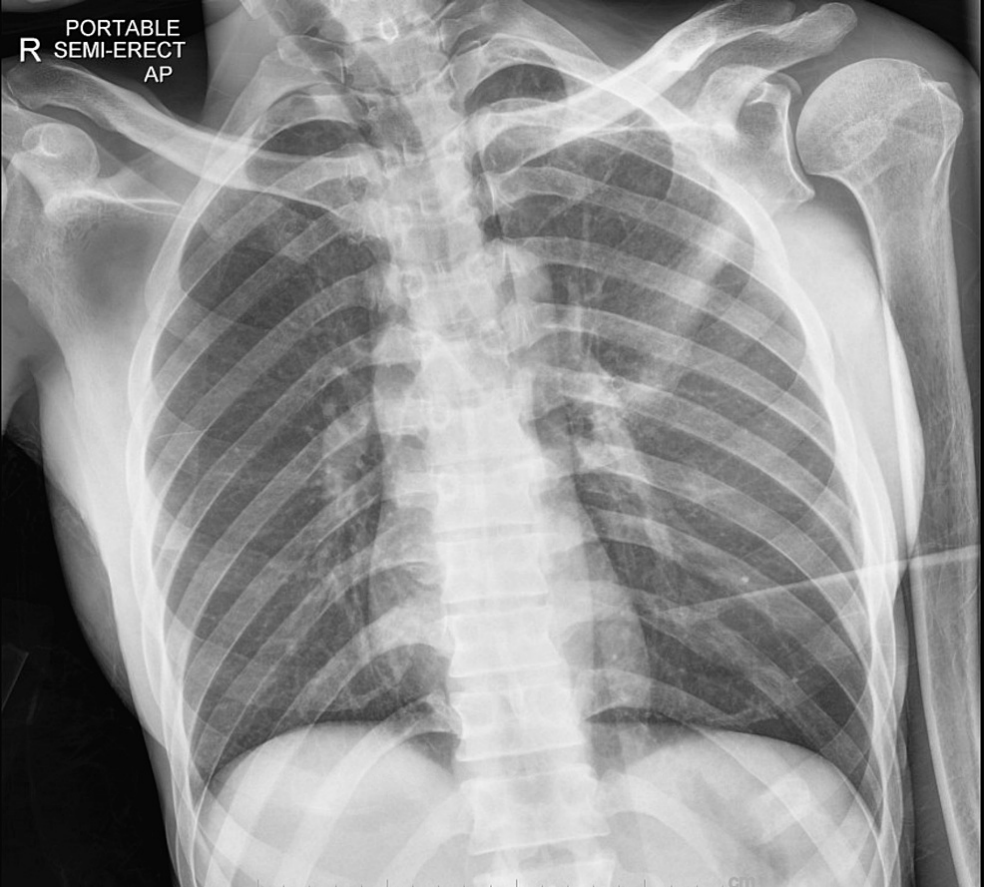
Chest X-ray at time of suspected IRIS, showing resolution of bilateral pulmonary infiltrate IRIS: immune reconstitution inflammatory syndrome

**Figure 4:**
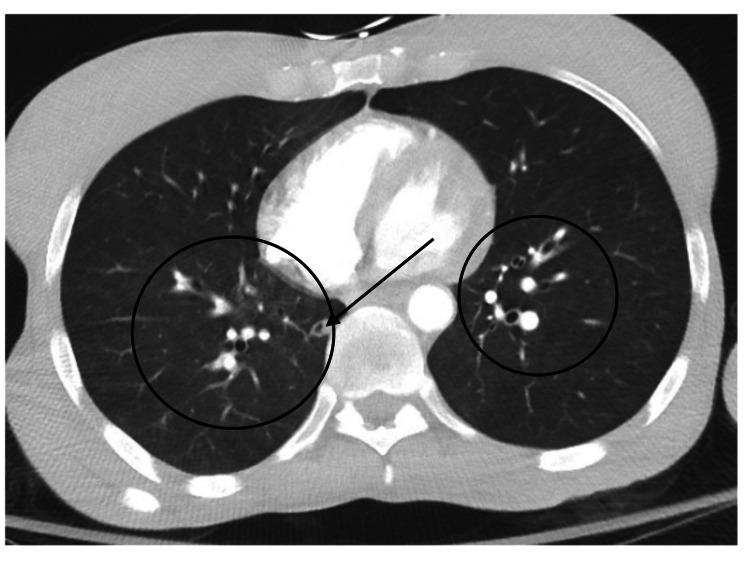
CT of chest at time of suspected IRIS, showing interval resolution of bilateral pulmonary infiltrate (circles) and stable cavitary lesion (arrow) IRIS: immune reconstitution inflammatory syndrome

Approximately five weeks after initiation of bictegravir, emtricitabine, and tenofovir, the patient developed leukocytosis (white blood cell count 172,000 with 81% neutrophils), she was experiencing malaise and fatigue and developed a fever with a maximum temperature of 102.4F. An electrocardiogram (ECG) showed sinus tachycardia. Lactic acid at this time was 2.5 mmol/L which downtrended to within normal limits after fluid resuscitation. She was started on prophylactic broad-spectrum antimicrobials, including vancomycin, cefepime, metronidazole, and acyclovir for possible hospital-associated infection. Another infectious workup was pursued. Blood cultures including fungal and AFB cultures were obtained and showed no growth (Table 1). Urinalysis was negative for infection. Chest x-ray showed resolution of previous interstitial opacities and no additional findings (Figure 3). CTA was negative for pulmonary embolism; it also showed improvement of bilateral ground-glass opacities and stable cavitary lesion, unchanged from prior imaging (Figure 4). In the absence of an infectious cause and due to the timing of the patient’s symptoms as it pertained to the initiation of anti-retroviral therapy, a diagnosis of IRIS was suspected. The patient’s symptoms resolved within two days of onset. She became hemodynamically stable for discharge and was ultimately placed in the skilled nursing facility.

## Discussion

Diagnosis of IRIS can be difficult due to the variety of presenting symptoms, patient complexity, and underlying etiologies. In our case, we had a patient who presented with advanced HIV with encephalopathy. Although the patient was treated for varicella encephalitis and MRSA meningitis, her cognitive function failed to improve to baseline. The patient might have developed irreversible encephalopathy secondary to underlying HIV-associated neurocognitive disorder. IRIS diagnostic criteria were met: the patient had a very low initial CD4 count with a high viral load (which is associated with increased prevalence of IRIS), developed inflammatory symptoms that were temporally associated with the initiation of ART, and no compelling evidence for another infectious or systemic cause of these symptoms were discovered despite investigation.

There is much that remains unknown and speculative about the pathophysiology of IRIS, but it is suspected that an underlying antigenic burden is acted upon by a recovering immune system to create the clinical manifestations of IRIS. The extent to which the clinical manifestations develop is thought related to the amount of antigenic burden present, the degree of immune restoration following ART initiation, and host genetic susceptibility [8,9]. The antigens that the immune system may act upon include both infectious and noninfectious agents [1,5,9]. When an infectious agent is suspected, it may have been previously diagnosed and treated, or it may have been subclinical and surface only when the host’s immune system recovers [9]. Non-infectious agents include many autoimmune pathologies such as systemic lupus erythematosus (SLE), rheumatoid arthritis (RA), thyroid disease, lymphoma, and others [9,10]. Initiation of ART appears to alter the concentrations of multiple pro- and anti-inflammatory cytokines and cause an increase in CD4+ and CD8+ T lymphocytes [3,11,12]. These immunologic changes are suspected to create a hyper-inflammatory state. 

In addition to the hyper-inflammatory signs previously discussed, additional symptoms of IRIS can also vary based on the underlying etiologies of IRIS. These additional symptoms are usually related to the worsening of the underlying disease process that is reactivated in patients with IRIS [10]. Thus, the clinical manifestations can be variable, which makes the diagnosis more challenging. There is also variety in the severity of the inflammatory syndrome that occurs, which may present on a spectrum between minimal symptoms and fatal progression of the disease [5]. Treatment of IRIS is targeted toward treating the underlying etiology; ART should be continued and with severe inflammatory symptoms, treatment with glucocorticoids or non-steroidal anti-inflammatory drugs (NSAIDs) can be considered [9]. Physicians must remain vigilant when presented with unexplained inflammation in patients with recent ART initiation as timely diagnosis and initiation of appropriate treatments may help decrease disease and symptom burden.

The limitation of this case report was the lack of repeat CD4 count and viral load. Though our patient did not have these repeated measures to indicate positive virologic response after initiation of ART, we still strongly suspect IRIS based on these other suggestive clinical factors. Due to the sheer number of infectious and non-infectious etiologies that can be implicated in IRIS, it is difficult to say with certainty what underlying etiology may have caused these symptoms. However, since varicella infection has been previously reported as an infectious etiology of IRIS [10,13], we suspect it is possible that the patient’s previous varicella infection could have been the underlying infectious burden that ultimately led to her IRIS symptoms and resolved with re-initiation of antiviral therapy.

## Conclusions

This case report contributes to the existing literature as an illustration of the IRIS thought to be induced by varicella infection. It additionally serves as a call to action to improve our recognition of IRIS in at-risk patients, standardizes our diagnostic criteria for IRIS in order to help increase recognition of those who suffer from this syndrome, and improves our understanding of the pathophysiology so we can develop therapeutic strategies to treat or avoid these complications.
